# Supplementary material for: Are carbon emissions trading and green financial instruments synergistic? -Comprehensive quantitative research based on content analysis
Source: PLoS One. 2024 Mar 7;19(3):e0298601. doi: 10.1371/journal.pone.0298601 (PMC10919601; doi:10.1371/journal.pone.0298601)
Supplement: S1 Data — (ZIP) [file pone.0298601.s001.zip › Data/地级市Z_F工作报告.zip!/║■▒▒/╢⌡╓▌╩╨/└┤╫╘╣1⁄2╓┌║┼í╛┬φ┐╦╩2╛▌═°í┐_2018.docx]

各位代表：

现在，我代表市人民政府向大会报告工作，请予审议，并请各位市政协委员和列席人员提出意见。一、2017年工作回顾

过去一年，是全面建设社会主义现代化国际航空大都市的开局之年。在市委坚强领导下，全市上下高举习近平新时代中国特色社会主义思想伟大旗帜，坚持以人民为中心的发展思想，坚定不移转方式、调结构，经济社会平稳发展，稳中向好。预计全年地区生产总值比上年增长8.2%，地方公共财政预算收入增长10.2%，规上工业增加值增长8%，固定资产投资增长15%，社会消费品零售总额增长12.8%，城镇居民人均可支配收入增长9.1%，农村居民人均可支配收入增长9%，大部分指标位居全省前列。

（一）经济结构调整稳中有进

服务业加速发展。服务业增加值占GDP比重提高3个百分点，突破36%，发展速度全省第一，三次产业结构进一步优化。服务业税收占比历史性超过工业，达到53%。电商企业总数突破30家，年销售额130亿元，增长40%，生产性服务业成为拉动第三产业增长的龙头力量。

工业提质增效。钢铁、水泥去产能591.2万吨。传统产业技改投入达103.2亿元，增长167.6%。规上工业企业主营业务收入、利润分别增长13.1%和14.7%。工业经济在强力去产能中实现稳健增长。爱民制药、枫树线业等技改项目投产，南都新能源、虹润新材料等重点产业项目试产，电厂三期稳步推进，工业发展后劲增强。荣获中国“质量之光魅力城市”称号，湖北名牌产品累计达57个。

农业结构调整加快。投入10.5亿元推进农业供给侧结构性改革。生态农业扩规提标，新增生态农业基地30家，总数达443家，新增“三品一标”认证30个，农产品质量安全检验检测合格率为99.5%。台湾杏福农业生态园、石竹生态园等田园综合体示范作用日益显现，农旅融合发展渐成新趋势。

科技创新成效明显。实施“新鄂州人”计划，引进和培育高层次人才1177名。加强与华科、华师、中科院武汉分院等高校院所合作，搭建创新平台。各类科技企业孵化器新增在孵企业85家，市校合作科技创新项目108个。高新技术产业增加值150亿元，增长15%；全社会研发投入占GDP比重达1.6%，万人发明专利拥有量2.23件。高新技术企业挂牌“新三板”累计达7家。

八大功能区竞相发力。坚持以产兴城、以城促产，积极推进沿江滨湖新区开放开发，打造产城融合新平台。八大功能区基础设施建设和产业集聚步伐加快，地区生产总值、固定资产投资、工业增加值等指标占全市比重超过60%，成为创新创业的主战场和高质量发展的重要支撑。

（二）大项目集中落地开工

湖北国际物流核心枢纽项目顺利动工。省、市与顺丰合作协议全部签订，机场合资公司成立。坚决落实省委、省政府要求，克服用地、招标、拆迁等难题，全面启动总投资180亿元的枢纽项目核心主体工程、走马湖水系治理、机场快速通道（S203）等综合配套工程建设。这些项目的集中开工，极大鼓舞了全市干部群众的信心和斗志！

大型基础设施项目密集推进。全市基础设施建设完成投资437亿元，增长25%。其中主城区完成投资43亿元，是上年的1.9倍。投资32.8亿元的文化、体育、会展三大中心开工，投资93亿元的鄂咸高速、投资58亿元的樊口公园、投资15.5亿元的三江港区综合码头一期工程等一批重大项目加快推进，武阳高速鄂州段顺利动工。抓住政策窗口期，创新建设投融资方式，有效解决了建设资金不足问题。

征地拆迁规范高效推进。出台集体土地房屋征收补偿安置办法等政策，统一征拆标准。全市拆迁近15000户，是上年的10倍，创历史新高。基本完成机场核心区拆迁工作，5个多月拆迁5000多户。新开工棚改项目21个、安置房35044套，是上年的2.3倍。被征迁群众顾大家、舍小家，离开世代居住的故土，所表现出来的顾全大局、甘于奉献的情怀，以及基层党员干部特别能吃苦、特别能战斗的工作作风，值得我们永远铭记。在此，我代表全市人民向他们表示衷心感谢！

（三）人民获得感显著增强

人居环境更新更优。投资3.8亿元创卫创文，全市人民齐心合力，国家卫生城市创建成功，全国文明城市创建增强了市民获得感。实施背街小巷改造、老城区雨污分流等工程，集中整治553个老旧小区，拆除违建亭棚722个，新装社区路灯1100盏，清除卫生死角，去除“牛皮癣”，疏通下水道，市容市貌得到较大改变。农村垃圾综合治理成效显著，改造旱厕3752所，推进21个“美丽乡村”试点建设。

城乡品质明显提升。新港路、马鞍山道路及地下综合管廊加快推进，吴都中央生态廊道、东洋澜湖湿地公园一期开放，凤凰大桥通车，市民中心竣工。洋澜湖激光水秀提升城市品位。光纤到村全覆盖，凤凰广场等公共场所开通免费公益WiFi。新建城东、太和水厂，改扩建雨台山水厂，改造供水管网，解决水压不足问题。实施公路安全生命防护工程。60台新能源公交车投入运营。在城区开展“两车”整治，实施交通文明工程，营造了安全有序的通行环境。

精准脱贫步伐加快。完成14个村、6449人的脱贫任务。加大财政投入，进一步推进产业扶贫、健康扶贫、金融扶贫、教育扶贫。发放小额信贷4230万元，扶贫对象医保全额补助，住院和重症慢性病门诊费用报销比例达90%，区级实行先诊疗后付费，困难群众满意度明显提高。

社会保障扩面提标。城市、农村低保标准分别提高到每月550元和400元，特困人员救助供养标准提高到每月740元，社会救助标准高于全省平均水平。在全省率先完成农村福利院“冬暖工程”。机关事业单位养老保险改革加快推进，实现城乡居民基本医保制度统一和一体化管理。实施社保“一卡通”工程。实行就业兜底保障，动态消除“零就业家庭”，城镇新增就业2.68万人。城镇登记失业率2.03%。

教文卫全面发展。改造50所义务教育薄弱学校，建设19所普惠性幼儿园。学生综合实践基地投入使用。鄂城区义务教育均衡发展通过国家评估复查。建设市图书馆新馆，新建基层文化广场100个，全运会金牌实现零的突破。加快推进健康鄂州建设，开展家庭医生签约服务试点，实现基本生育免费服务全覆盖。全面取消以药养医，公立医院药品价格大幅下降。市精准医疗中心和省中医院武东院区主体大楼封顶。

（四）生态文明建设成效显著

长江大保护行动力度空前。铁腕开展长江饮用水水源地环境大整治，拆除长江干线码头114个，拆除水源地保护区内房屋221户，拆迁“散乱污”企业110家，复绿长江滩涂1000亩。拆除了多年想拆除却拆不动的码头砂站和污染企业，还长江岸线于自然，切实保障了市民饮水安全。

湖泊生态修复深入推进。全面完成梁子湖湿地保护区71处环保问题整改。拆除2.2万亩湖面围网，取缔1.3万亩珍珠养殖。完成畜禽养殖“三区”划分，取缔禁养区90家养殖场。落实四级河湖（库）长制度。投资8亿元实施青天湖水生态修复、洋澜湖生态补水和曹家湖、垱网湖退田还湖工程。

环境治理保持高压态势。全面完成中央环保督察整改任务。完成葛华污水处理厂整治和花湖、红莲湖、梁子岛污水处理厂建设。实施余寿港、丁桥港、五丈港黑臭水体综合治理。系统治理工业烟粉尘、秸秆焚烧、机动车尾气、餐饮油烟等大气污染源，空气优良天数上升10.8%。

（五）社会治理开创新局面

强力开展“霹雳行动”，打掉违法犯罪团伙262个，“黑恶霸、黄赌毒、盗抢骗”以及邪教、“三堵五闹”等违法犯罪活动受到严厉打击，人民群众安全感大幅提升，信访维稳秩序明显改善，全国全省重大节会、重要活动期间鄂州平安稳定。严守安全生产红线，事故发生率下降57.1%。连续17年未发生重大食品药品安全事故。深化商事制度改革，实行三十二证合一，加强事中事后监管，创新消费维权模式。继续推进“一村（社区）一律师”工作。支持深化国防和军队改革，海军“鄂州舰”入列。

我们不断加强政府自身建设，把全面从严治党要求贯穿政府工作各方面，认真贯彻中央八项规定精神，夯实主体责任，驰而不息正风肃纪，干部作风有所改进。法治政府建设成效明显，依法接受市人大及其常委会的法律监督和工作监督，主动接受市政协的民主监督，共办理人大建议、政协提案366件。

外事、统计、审计、编制、保密、工会、档案、气象、新闻出版广电、民族宗教、防灾减灾、对外援助、妇女儿童、老龄、残联、社会科学等各方面工作都取得了新成绩。

各位代表，过去一年的成就，是我们认真学习贯彻习近平新时代中国特色社会主义思想的结果，是市委科学决策、坚强领导的结果，也是全市人民共同奋斗的结果。

　　在此，我代表市人民政府，向各位人大代表、政协委员，向各民主党派、工商联、无党派人士、人民团体，向驻鄂州部队官兵，向所有参与、支持、关心鄂州建设发展的同志们、朋友们，表示衷心感谢和崇高敬意！

同时，我们也清醒地认识到，发展中的困难和挑战不少。我市经济总量偏小，结构调整和动能转换任重道远；民生领域还有不少短板，城市管理长效机制尚不健全；城市大建设和产业大发展面临空间、人才、土地和资金等瓶颈制约；建设社会主义现代化国际航空大都市对政府治理体系和治理能力提出了新的更高要求，等等。对于这些问题，我们必须高度重视，认真解决。二、2018年工作安排

2018年是贯彻党的十九大精神的开局之年。在习近平新时代中国特色社会主义思想指引下，市委七届五次全会作出全面建设社会主义现代化国际航空大都市的战略部署。我们对历史方位的把握、战略部署的安排和发展路径的规划从来没有像今天这样如此清晰，我们比历史上任何时期都更接近、更有信心和能力实现城市现代化目标。我们要牢牢把握高质量发展这个根本要求，以敢为人先、时不我待的担当和气魄加快推动城市转型发展，在新一轮区域竞争中抢占制高点、赢得主动权。

今年政府工作的总体要求是：全面贯彻党的十九大精神，以习近平新时代中国特色社会主义思想为指导，加强党对经济工作的领导，坚持稳中求进工作总基调，坚持新发展理念，紧扣我国社会主要矛盾变化，按照高质量发展的要求，统筹推进“五位一体”总体布局和协调推进“四个全面”战略布局，坚持以供给侧结构性改革为主线，以社会主义现代化国际航空大都市建设为主战场，统筹推进稳增长、促改革、调结构、惠民生、防风险各项工作，优先解决人民最关心最直接最现实的利益问题，着力推动事关长远的重大生产力布局和基础设施建设，着力推动事关全局的重大产业项目落地，着力推动城市治理体系和治理能力现代化，再创鄂州速度、效率、标准、质量，打好防范化解重大风险、精准脱贫、污染防治攻坚战，打造湖北特色产业增长极，促进经济社会持续健康发展，为湖北加快“建成支点、走在前列”、全面建设社会主义现代化强省作贡献、打头阵。

今年预期地区生产总值增长8%左右，地方公共财政预算收入增长8%，高新技术产业增加值占GDP比重17.5%，空气优良天数占比78%，城乡居民收入与经济增长同步。重点抓好以下八个方面工作：

（一）全面推进国际物流核心枢纽建设

围绕大飞机2020年从鄂州起飞的战略目标，倒排工期，挂图作战，争分夺秒加快空港建设，高起点发展现代枢纽经济，重塑发展格局。

加快机场核心区建设。按照港、产、城联动发展思路，完成全域城乡总体规划修编和重点区域控制性详规。创新统筹方式，实现机场核心区基础设施一次性规划、一体化设计、集成化建设。积极协助机场公司推进机场可研报告报批，配合推进机场总规和初步设计获批。全力支持机场建设，全面推进机场土石方、花马湖综合防洪排涝、机场500千伏超高压配电等系列工程，完成机场净空保护电磁监测站建设。

同步建设机场配套区域。稳步推进空港中央商务区建设，加快文化体育会展三大中心、美术馆、科技馆建设，启动周边中央公园、配套路网、商务及服务配套设施综合设计及工程实施。全力推进燕矶、杨叶、鹰山、月陂安置小区建设。持续推进机场快速通道（S203）等交通基础设施建设。

超前谋划现代枢纽经济。加强城市发展战略研究，高水平高质量编制现代空港经济发展规划。依托机场、三江港打造“铁水公空”多式联运体系，提升空港枢纽功能。开展鄂州航空都市区国际路演推介活动，提升鄂州空港全球影响力。围绕航空制造、电子信息、智慧物流、先进制造、供应链管理等领域谋划布局一批产业链长、带动力强的临空偏好型产业。

做好被征地农民市民化工作。以空港物流、临空制造和城市服务业为重点，因人制宜开展就业培训，确保有劳动能力的被征地农民充分就业。健全政策体系，解决被征地农民业教保医问题。提高集中安置区社区管理服务水平，让被征地农民安居乐业。

（二）加快建设现代化产业体系

以供给侧结构性改革为主线，坚持有所为有所不为，坚持“破”“立”“降”并举，加快建设实体经济、科技创新、现代金融、人力资源协同发展的高质量产业体系。

举全市之力引进行业龙头企业。精准定位，一企一策，克服空间小、成本高等制约，引进大项目、好项目。以政府产业基金为引领，统筹市场资源，组建空港产业基金，以资本纽带促进产业集群发展。葛店开发区、鄂州开发区分别引进1个100亿元和50亿元以上单体项目。鄂城区、华容区各引进1个10亿元以上单体项目。梁子湖区在亿元现代服务业项目引进上实现新突破。完成省外资金到位270亿元。

有扶有控调整产业结构。明确重点产业扶持政策，完善负面清单，将各类政策性财政资金打捆设立产业发展资金，精准有效支持产业发展。严格实行项目投资强度和土地双控，节约集约用地。推动从总量性去产能向结构性优产能转变，持续推进钢铁、水泥去产能。以生物医药、新能源、高端装备、金刚石刀具、工程塑胶等为重点，加强技术改造。大力发展民营经济，增强实体经济吸引力和竞争力，新增产值10亿元以上企业3家，新增规上企业30家。推进质量强市建设，鼓励企业争创长江质量奖，实施商标品牌战略。

提升服务业发展水平。突破性发展生产性服务业，推动电子商务线上线下全产业链融合发展，推进国家高技术检验检测服务业集聚区、华中冷链港等项目建设。持续优化生活性服务业，引进新业态，盘活城区闲置低效商业综合体，控制低端增量。推进恒大童世界项目建设，支持梁子湖旅游加快转型升级。

加快建设创新型城市。建立健全以企业为主体，市场为导向，产学研深度融合的技术创新体系，培育、引进一批创新型领军企业，支持中小企业创新。创新推进“121”人才池、“333”高层次创新创业人才计划，培养引进高水平科技创新团队和高素质“新鄂州人”。加快吸引一批国内外知名企业进驻，共建企业工研院，推动与中科院合作实质性落地，加快建设华师鄂州研究院。提升华科鄂州工研院、梧桐湖科技创意园等创新平台服务功能。实现科技创新重点突破和领域拓展，促进创新资源转化为现实生产力。探索科技与金融深度对接的新机制，为科技型企业提供全生命周期的金融服务，建立健全科技创新工作机制和考核机制，形成一系列政策工具箱，激发大众创新创造创业活力。

（三）推动区域协调发展

以社会主义现代化国际航空大都市建设统筹沿江滨湖新区开发开放，突出差异化发展，优化主体功能区布局，打造武鄂黄黄区域特色产业增长极。

加快形成差异化发展格局。葛店开发区打造中部地区有影响力的光电子信息产业集群和电子商务中心，加快产城融合，建设武鄂协同发展示范区。机场核心区、鄂城新区和花湖开发区连片开发，重点发展临空产业、现代服务业，打造空港经济核心区。三江港新区建设现代化物流新城，红莲湖新区建设文化旅游度假区。梁子湖区建设全国生态文明示范区，梧桐湖新区建设生态科学城。鄂州开发区建设高技术服务业和高端制造业聚集区。探索建立与区域特色及发展阶段相适应的财力性转移支付制度和台账考核制度，调动市区两个积极性。

推进武鄂协同和鄂黄黄联动发展。突出产业协同、城市规划、交通基础设施和生态环保，实现与周边城市协同发展。推动新城大道、高新四路与武汉东湖高新区道路对接。推进武汉地铁11号线至葛店项目开工。加快鄂咸高速、武阳高速鄂州段建设。以空港产业跨区域协同发展为重点，促进鄂黄黄城市联动发展，建立常态化工作联系机制。

（四）提升城市建设和管理水平

坚持市民角度、家园情怀、人本价值，尊重城市建设发展规律，统筹规划建设管理，提升城市品质。

系统建设城市基础设施。科学编制各类专项规划。实施道路升级工程，推进马鞍山道路、孙权大道及地下综合管廊建设，加快新港路、凤凰路交通综合整治等工程建设。实施生态景观工程，推进樊口公园、滨江风光带四期、东洋澜湖生态修复、主城区雨污分流改造等项目建设。实施公用设施工程，推进主城区供水管网改造，建成城南、庙岭、燕矶等加压站，完成雨台山水厂改扩建和城东水厂建设。做好5G基站规划布点。加快临江220千伏、姚家湖110千伏输变电工程进度。推行殡葬改革，加快长山、团山公墓建设。

加强城市精细化管理。巩固创卫成果，常态化创建文明城市。探索主城区环卫一体化，推进作业标准化。率先在机关事业单位推行垃圾分类。改革主城区征收拆迁工作机制，理清部门职责，落实属地责任，重拳打击违法建设。加强网约车、共享单车监管。严格交通管理，畅通微循环，消除一批堵点。实施城市照明和亮化工程。严格执行养犬管理等政府规章。

加强和创新社会治理。统筹城市管理和社会治理，推动服务与执法重心下移，推进社会治理共建共治共享。理顺街办、社区关系，让社区回归为民服务本源。改造升级社区党员群众服务中心。完成村（居）委会换届选举。大力培育社会组织，创新“三社”联动机制，实现政府治理和社会调节、居民自治良性互动。推进志愿服务制度化。深入推进平安鄂州建设，完善社会矛盾预防化解体系。推进“霹雳行动”常态化，持续严打“黑恶霸、黄赌毒、盗传骗、食药环”等违法犯罪活动，大力推进立体化、信息化社会治安防控体系建设。深入开展“七五”普法。完善双重预防性工作机制，夯实安全生产基层工作和监管责任，有效遏制各类事故，实现安全生产“一无一降”目标。强力开展货车超限超载治理工作。强化市场监管，加强信用体系建设，维护消费者权益。实施食品安全战略，让群众吃得放心。

（五）建设美丽鄂州

持续推进长江大保护九大行动，更多用法治方式解决历史遗留问题，用市场手段探索可持续发展路径。

加大生态系统保护力度。修复湖泊湿地生态，加快樊口二站等水利补短板项目建设，完成洋澜湖生态补水、红莲湖-大头海连通和曹家湖、垱网湖退田还湖等工程。创建梁子湖湖泊治理国家示范区，推进《梁子湖省级湿地自然保护区规划》修编工作。全面开展梁子湖、花马湖禁捕工作。构建生态廊道和生物多样性保护网络。提升江河湖库水质，综合整治入江排污设施，关闭燕矶、临江自来水厂，对重点水域开展实时在线监测，严格落实河湖（库）长制度。创建森林城市，绿化荒山3600亩，完成长江沿线及江心洲绿化防护林1000亩。

着力解决突出环境问题。巩固中央环保督察问题整改成果，对违法行为“零容忍”。确保花湖、红莲湖等集镇污水处理厂达标运行，推进村湾污水处理设施建设，基本实现城乡污水全收集全处理全覆盖。开展第二次全国污染源普查和土壤环境详查。提高钢铁、水泥地方环保标准，稳妥有序倒逼重点污染企业退出。着力治理扬尘、机动车尾气等污染源，打赢蓝天保卫战。启动餐厨垃圾、秸秆等资源化利用项目建设。

深入实施生态价值工程。推进自然资源资产负债表编制和领导干部自然资源资产离任审计，加强成果应用。开展第三次全国土地调查。核算生态价值，继续实施以生态价值补偿为核心的财力性转移支付。与国家开发银行湖北分行等机构合作深化绿色金融改革，创新生态资产质押、收益权转让等融资方式。

（六）实施乡村振兴战略

突出生态农业发展和农村人居环境改善，统筹空间规划、基础设施、公共服务、生态环保、要素流动，促进农业农村现代化。

推动农业现代化。深化农业供给侧结构性改革，坚持生态农业主题不变，加快生态农业基地提档升级，实现质量兴农、绿色兴农。落实粮食安全行政首长负责制。支持农产品公共品牌建设，促进“三品一标”农产品做精做优。大力发展休闲体验农业，建设一批有全省影响力的田园综合体。加快培育农民专业合作社等新型农业经营主体，健全农业社会化服务体系，着力破除小农户与现代农业衔接障碍。

实施“三乡工程”。制定“乡村振兴”总体方案，明确空间布局、主要任务和实施路径，出台支持政策，鼓励“市民下乡、能人回乡、企业兴乡”，盘活农村闲置房屋和土地。深入开展农村人居环境整治，完善农村垃圾综合治理长效机制，完成21个省、市级美丽宜居示范村建设。推进“四好农村路”建设，新建100公里通村公路。加强农村基层基础工作，健全自治、法治、德治相结合的乡村治理体系。推进集镇改造升级，创建示范性特色小镇。

深化农村产权制度改革。巩固农村基本经营制度，完善承包地“三权分置”制度，着力探索规范有效的土地集中流转模式。深入推进农村集体产权制度改革，完成农村集体资产清产核资、清人分类工作，对有经营性资产的村开展集体资产股权化改革，发展壮大村级集体经济。加快“房地一体”的农村宅基地和集体建设用地确权登记颁证工作，重点突破农村产权抵押贷款难题。

（七）切实保障和改善民生

从群众最直接最迫切的问题着手，既尽力而为，又量力而行，一件接着一件办，一年接着一年干，不断满足人民日益增长的美好生活需要。

强化就业和社会保障。实施就业兜底保障和创业促进计划，缓解结构性就业矛盾，开发村组公益岗位2000个，新增城镇就业1.9万人，城镇登记失业率控制在4%以下，建成市就业服务中心。加强农民工工资清欠工作。深入推进养老保险制度改革，执行最低生活保障和特困人员供养标准。推动社区养老、医养结合，加强对留守儿童、残疾人等弱势群体帮扶。完善促进房地产市场平稳健康发展的长效机制，实行分区域差别化调控政策，加大棚户区改造力度，加快城南、城东安置小区建设。

提高公共服务供给质量。坚持教育优先发展，强化素质教育，着力解决城区大班额、择校热问题。新建、改扩建10所农村公办幼儿园、40所义务教育薄弱学校。优化学校布局，加快机场配套区域学校、城东学校、市二中、葛店高中、华师梁子湖校区等建设。加强基层文化阵地建设，实施吴都生态廊道体育健身区、全民健身中心、环梁子湖生态绿道等工程，打造文化体育品牌，提升文化发展软实力。实施健康鄂州工程，深化医药卫生体制改革。推进乡村卫生一体化管理，提升基层医疗卫生服务质量。建设儿童医院及城东、城南社区卫生服务中心，市精神卫生中心投入使用。落实全面两孩政策，促进人口均衡发展。

打好脱贫攻坚战。充分发挥政府、市场和社会协同作用，精准扶贫，精准脱贫。加大产业扶贫、健康扶贫、金融扶贫、教育扶贫等政策落实力度。开展脱贫攻坚“作风建设年”活动，进一步压实各级干部结对帮扶责任，确保6个省级贫困村出列，1万名以上贫困人口脱贫。

解决群众关切的热点问题。整治和改造老旧小区，规范物业管理。加强电梯安全监管，规范小区废旧回收，着力解决下水道堵塞、油烟、噪音扰民等问题。全力推进“厕所革命”。新增60辆新能源公交车，优化公交线路。规范出租车营运，解决违规强行拼客、服务态度差等问题。多措并举，逐步缓解老城区停车难问题。加强房地产市场监管，分类分批解决不动产证“办证难”问题。

（八）深化改革扩大开放

着眼全局，以改革破解难题、激发活力，以开放拓展空间、培育动力，厚植转型发展新优势。

推进重点领域改革。完善土地储备和出让机制，提高耕地占补平衡开发投入，精准供地，严格土地执法，最大力度盘活闲置低效土地。深化行政审批制度改革，推进商事制度改革，推行“马上办、网上办、一次办”政务服务机制，倒逼干部作风转变，优化营商环境，构建“亲清”政商关系。按照“警务前移、实战实用、专综统筹、扁平高效”方向，全面深化公安改革。建立部门预算制度和财政支出绩效考核制度，提高预算科学性和资金使用效率。深化投融资体制改革，推进城投公司市场化运作，提高土地和金融资源统筹能力。防范化解政府债务风险，确保政府负债率不超过全省平均水平。统筹全市信息化建设，整合市直单位办公楼资源，提高资源使用效率。深化城管执法体制改革和国有企业改革。推进市审计、广电等领域体制改革。

提升内陆开放水平。积极申报国家级空港经济试验区，加强与湖北自贸区武汉片区的工作对接，为纳入扩展范围做好准备。推进三江港联检大楼建设，同步规划建设机场口岸。瞄准世界枢纽经济城市，开展国际交流与合作。三、加强政府自身建设

不忘初心、牢记使命。我们要不断增强本领、转变作风，加快建设依法行政、务实高效、勤政为民、人民满意的服务型政府。

坚持党的领导。认真学习贯彻党的十九大精神，以习近平新时代中国特色社会主义思想武装头脑。坚决维护以习近平同志为核心的党中央权威和集中统一领导，自觉在思想上政治上行动上同党中央保持高度一致。把省委、省政府和市委的各项决策部署落实到政府工作全领域、全过程。

坚持依法行政。加强法治政府建设，建立健全制度体系。完善矛盾纠纷化解机制，深入推进行政复议体制改革。自觉接受党内监督、人大监督、民主监督、国家监察，完善社会监督和舆论监督机制。提高政务公开信息化、集中化水平。

坚持清正廉洁。落实全面从严治党主体责任，严格履行“一岗双责”，扎实推进党风廉政建设，坚持反腐败无禁区、全覆盖、零容忍。严格贯彻落实中央八项规定实施细则精神，深入查摆“四风”，突出整治“表态多调门高，行动少落实差”等10种形式主义、官僚主义表现，持续推进作风建设常态化、长效化。

坚持务实重行。坚持“为人民办事、对人民负责、向人民报告、让人民满意”，集中财力解决人民最关心、最直接、最现实的利益问题。坚持开短会、说实话、办实事。狠抓工作落实，对年度重点目标任务实施台账管理，四位一体安排项目立项、资金保障、实施方案和督办考核。加强学习，大兴调查研究之风，培养专业能力，提高政府科学决策、破解难题的水平。增强执行力，强化督查问责，坚决整肃庸政懒政怠政行为。

各位代表，使命呼唤担当，使命引领未来。让我们高举习近平新时代中国特色社会主义思想伟大旗帜，在省委、省政府和市委的坚强领导下，以永不懈怠的精神状态和一往无前的奋斗姿态，保持政治定力，坚持实干兴邦，为把鄂州建成社会主义现代化国际航空大都市而努力奋斗！
